# Long Telomeres in Blood Leukocytes Are Associated with a High Risk of Ascending Aortic Aneurysm

**DOI:** 10.1371/journal.pone.0050828

**Published:** 2012-11-29

**Authors:** Tuija J. Huusko, Merja Santaniemi, Sakari Kakko, Panu Taskinen, Olavi Ukkola, Y. Antero Kesäniemi, Markku J. Savolainen, Tuire Salonurmi

**Affiliations:** 1 Institute of Clinical Medicine, Department of Internal Medicine, Clinical Research Center, Oulu University Hospital and University of Oulu, Oulu, Finland; 2 Biocenter Oulu, University of Oulu, Oulu, Finland; 3 Institute of Clinical Medicine, Department of Surgery, Oulu University Hospital and University of Oulu, Oulu, Finland; Innsbruck Medical University, Austria

## Abstract

Ascending aortic aneurysm is a connective tissue disorder. Even though multiple novel gene mutations have been identified, risk profiling and diagnosis before rupture still represent a challenge. There are studies demonstrating shorter telomere lengths in the blood leukocytes of abdominal aortic aneurysm patients. The aim of this study was to measure whether relative telomere lengths are changed in the blood leukocytes of ascending aortic aneurysm patients. We also studied the expression of telomerase in aortic tissue samples of ascending aortic aneurysms. Relative lengths of leukocyte telomeres were determined from blood samples of patients with ascending aortic aneurysms and compared with healthy controls. Telomerase expression, both at the level of mRNA and protein, was quantified from the aortic tissue samples. Mean relative telomere length was significantly longer in ascending aortic aneurysm blood samples compared with controls (T/S ratio 0.87 vs. 0.61, p<0.001). Expressions of telomerase mRNA and protein were elevated in the aortic aneurysm samples (p<0.05 and p<0.01). Our study reveals a significant difference in the mean length of blood leukocyte telomeres in ascending aortic aneurysm and controls. Furthermore, expression of telomerase, the main compensating factor for telomere loss, is elevated at both the mRNA and protein level in the samples of aneurysmal aorta. Further studies will be needed to confirm if this change in telomere length can serve as a tool for assessing the risk of ascending aortic aneurysm.

## Introduction

Telomeres are tandem repeats of a specific DNA sequence (TTAGGG) at the ends of chromosomes. Telomere shortening is a natural, usually age related phenomenon which occurs with every cell division. Still, when telomeres have become critically shortened, the cells become senescent and apoptosis may be triggered. The mean telomere length varies already between newborns since it is partly genetically inherited. However, environmental factors, such as oxidative stress, inflammation and increased cell turnover associated with cardiovascular risk factors have a role in attenuating the telomere length during an individual’s life [1].

Telomerase (TERT) enhances the addition of tandem repeats to telomeres and this enzyme is widely expressed in the embryonic stem cells. TERT activation is weakened in mature cells during aging, leading to telomere shortening. Expression of TERT is inhibited at the gene level by histone deacetylation and DNA methylation whereas its activation takes place in areas where cells are undergoing rapid expansion. In somatic cells, telomeres shorten with each cell division since DNA polymerase is incapable of completing the replication of the 3′ end of linear DNA molecules. TERT should serve as a protective capping of linear DNA, but in the majority of adult somatic cells, telomerase activity is deficient [2].

The biological aging of the aortic wall may have an important role in the pathophysiology of aortic aneurysm [3]. In general, ascending aortic aneurysms (AscAAs) are a consequence of the connective tissue destruction; one common histopathological finding is the loss of smooth muscle cells caused by degeneration of elastic and collagen fibers. The pathogenesis of AscAAs is rarely associated with atherosclerosis and it is commonly genetically inherited [4]. AscAA research has focused on molecular biology studies of aortic tissue and on association studies of various chromosomal loci. There can be mutations in several of the genes responsible for AscAAs for example ACTA2, MYH11, TGFBR1, TGFBR2 and SMAD3 [5]–[9]. Recent studies have shown that a short blood leukocyte telomere length (LTL) is associated with aortic aneurysms [10]–[12]. Therefore, we have investigated whether the relative LTLs are changed in AscAAs as compared with age-matched controls and furthermore, whether the hTERT (h, human) expression is altered in the aortic tissue samples of AscAAs compared with control tissues.

## Materials and Methods

### Ethics

The study was approved by the ethical committee of Oulu University (17.6.1998) and ethical committee of Oulu University Hospital (53/2002 and 17.12.2007). Patients who provided their written informed consent were included in this study.

### Study Subjects

Patients diagnosed with AscAA during the years 1996–2007 in the Oulu University Hospital were recruited and those who provided informed consent were included in this study. Patients with AscAA related to a thoracic trauma or to a previous aortic operation and patients with Marfan syndrome were excluded. Blood samples from a total of 86 AscAA patients were collected for the relative LTL study. A total of 86 blood samples of subjects without any diagnosis of aneurysmal disease (i.e. known own or family history with aortic aneurysms) were selected from our control population samples that has been collected randomly from the population of the Oulu area with the help of the Population Register Centre. Control samples were age and gender matched. Samples of the aortic tissues intended for the quantitative real-time PCR (RT-qPCR) and immunostaining study were collected during the aortic or coronary bypass operations at the Oulu University Hospital. A total of 29 ascending aortic aneurysm tissues and 14 control tissues were collected and analysed with the RT-qPCR method for hTERT expression. Control aortic tissues were taken during coronary bypass surgery from an area without any macroscopic atherosclerotic changes of non-dilated thoracic ascending aorta. In the hTERT immunohistochemistry, 12 AscAA and 5 control tissues were used.

### Quantitative PCR Analysis for Blood Leukocyte Relative Telomere Length

The relative lengths of the telomeres were estimated by the quantitative PCR (qPCR) method according to Cawthon [13]. This method allows the determination of the telomere content of DNA against single copy gene. Genomic DNA was extracted from the blood samples and stored at 4°C at a concentration of 250 ng/ml until qPCR analysis. Plasma lipoprotein fractions were separated by sequential ultracentrifugation and concentrations of cholesterol and triglycerides in plasma were determined by enzymatic colorimetric methods, as described earlier [14].

PCR of the telomere DNA was carried out by using iQ™ SYBR® Green Supermix (Applied Biosystems, Foster City, CA, USA) performed with IQ5 Real-Time PCR Detection System (Biorad, Hercules, CA, USA). Telomeric DNA was amplified by using Tel-1: GGTTTTTGAGGGTGAGGGTGAGGGTGAGGGTGAGGGT and Tel-2: TCCCGACTATCCCTATCCCTATCCCTATCCCTATCCCTA primers. For the amplification of the control gene, the following acidic ribosomal phosphoprotein 36B4 primers were used: 36B4u: CAGCAAGTGGGAAGGTGTAATCC and 36B4d: CCCATTCTATCATCAACGGGTACAA. Reagents for qPCR were 5 ng of template DNA and primers Tel1∶200 nm and Tel2∶675 nm or 36B4u: 300 nm and 36B4d: 500 nm combined in SYBR® Green Supermix, final volume of 25 µl for each reaction. All reactions were assayed as triplicates and patient and control samples were analysed side by side on the plates. A serially diluted standard curve from 3.125 ng to 12.5 ng of the reference DNA was included in each assay. The profile of the telomere amplification was: 95°C for 10 min, 30 cycles of 95°C for 15 s, 54°C for 2 min. For 36B4 amplification, the PCR conditions were 95°C for 15 min, 40 cycles of 95°C for 15 s and 60°C for 60 s. Relative expressions of LTL in genomic samples were calculated according to Cawthon [13] and the standard curve was used for relative quantitation. Results are shown as T/S ratio, where T is the amount of telomere and S is the amount of a single copy gene in each well.

### Quantitative Real-time PCR for Telomerase

Aortic tissues were snap frozen in liquid nitrogen and stored at −80°C until RNA extraction. RNAs from the aortic tissues were extracted using Trizol® reagent (Invitrogen, Carlsbad, CA, USA). Briefly, 50–100 mg of the tissue was homogenized in 1 ml of Trizol reagent and mRNA was extracted according to the manufacturer’s instructions. The extracted RNA was purified with RNeasy Mini Kit (Qiagen Inc, Valencia, CA, USA).

cDNAs of the aortic tissues of AscAAs and controls were synthesized from the mRNA by cDNA First Strand Synthesis Kit (MBI Fermentas, Heidelberg, Germany). RT-qPCR was performed with IQ5 Real-Time PCR Detection System (Biorad, Hercules, CA, USA) by using the following primers for TERT (2673U: TGACACCTCACCTCACCCAC, 2767L: CACTGTCTTCCGCAAGTTCAC, Sigma-Aldrich Co., St. Louis, MO, USA). Gene expression was quantified by using the SYBR Green PCR Master Mix Kit (Applied Biosystems, Foster City, CA, USA). Each sample was analysed as duplicates and an interrun calibrator, negative control samples and the standard curves were included in each experiment. The RT-qPCR conditions were: 95°C for 3 min; 40 cycles at 95°C for 10 s; 58°C for 10 s; and 72°C for 10 s. GAPDH (glyceraldehyde 3-phosphate dehydrogenase) gene was used as an endogenous control. The amount of mRNA for each sample was normalised using the average of the pooled sample which was included in all plates. A melting curve analysis was included at the end of every run. Expression levels were estimated by the normalized expression method (ΔΔCt) according to the manufacturer's instructions (Biorad).

### Immunostaining of Aortic Tissue Samples with Telomerase Antibody

Paraffin-embedded sections were stained as described earlier [15]. Briefly, paraffin-embedded sections (4 µm) of the aortic tissue were stained with the DAKO envision immunostaining method (DAKO, Glostrup, Denmark). Firstly, paraffin sections were deparaffinised, rehydrated and unmasked with a high temperature and EDTA (ethylenediaminetetraacetic acid) treatment. DAKO-blocking solution was used for the blocking reaction of slides before addition of the primary antibody against hTERT (TERT-Y707, Aviva Systems Biology Corp., San Diego, CA, USA). The slides were incubated with the dilution of 1∶100 of hTERT antibody for 30 minutes followed by 30 min incubation with a secondary antibody (DAKO). The binding reaction was detected using DAB (3,3′-diaminobenzidine) (DAKO) and the slides were counterstained with hematoxylin-eosin. The results of the immunostaining study were analysed in a Leica DM 3000 microscope and stained areas were quantified with Leica Qgo and QWin morphometric programs (Leica, Heerbrugg, Switzerland).

### Statistical Analysis

Differences between study groups were analysed with t-test or Fisher’s exact test. Multivariate odds ratios (OR) with 95% confidence interval (CI) for OR were calculated by using logistic regression analysis. Partial correlation analysis was used to test for correlation. RT-qPCR results are presented as a mean of normalized expression and SD. Results were analysed with IBM SPSS Statistics (version 20 for Windows) (SPSS, Chicago, IL, USA).

## Results

### Study Subjects

Table 1 summarizes the characteristics of the study population. The AscAA patients matched well with healthy controls in terms of age and sex. The mean ages (± standard deviation, SD) of the AscAA patients and healthy controls were 58 years for both groups (±9 and ±5, respectively).

**Table 1:** Demographic characteristics of the study groups in the relative telomere length analysis.

Variable	AscAA	Control	P-value
Total n	86	86	
Age (years)	58 (±9)	58 (±5)	
Male	68 (79%)	67 (78%)	
BMI (kg/m^2^)	28 (±5)	27 (±4)	NS
Total-cholesterol (mmol/l)	5.3 (±0.9)	5.4 (±1.3)	NS
HDL-cholesterol (mmol/l)	1.1 (±0.3)	1.5 (±0.5)	<0.001
LDL-cholesterol (mmol/l)	3.5 (±0.9)	3.6 (±1.1)	NS
Triglycerides (mmol/l)	1.6 (±0.9)	1.2 (±0.5)	<0.001
Hypertension	63 (73%)	28 (33%)	<0.001
CHD	22 (26%)	8 (9%)	<0.01
Diabetes	10 (12%)	4 (5%)	NS
Smokers (*ex-smokers and current*)	43 (50%)	52 (61%)	NS
Use of statins	27 (31%)	16 (19%)	NS

Mean age in years, BMI, total-cholesterol, HDL-cholesterol, LDL-cholesterol and triglycerides with standard deviation are shown. For other variables, number of subjects (percentage) is shown. *AscAA*, ascending aortic aneurysm, *BMI,* body mass index, *CHD,* coronary heart disease, *NS*, not statistically significant.

Plasma HDL-cholesterol levels were significantly lower (p<0.001) and triglyceride levels higher (p<0.001) in the samples of AscAA patients when compared with controls. Furthermore, the AscAA group was more likely to suffer from hypertension and CHD (coronary heart disease) (p<0.001 and p<0.01) than the control group.

### Relative Leukocyte Telomere Length Analysis

Relative LTLs were measured from a total of 172 DNA samples. AscAA patients had significantly longer relative LTLs than their healthy age matched controls, i.e. the T/S ratio 0.87 vs. 0.61 (p<0.001) (Table 2). The length of telomeres was the same in men and women (p = 0.178) and statin use had no effect on relative LTLs (p = 0.946). Correlation analysis revealed a significant inverse correlation between relative LTL and age (r = −0.250, p<0.01 for both groups, r = −0.260, p<0.05 for AscAAs and r = −0.230, p<0.05 for controls). Furthermore, a significant inverse correlation between the relative LTL and plasma triglycerides was found in control group (r = −0.234, p<0.05). No significant correlations for other variables, i. e. plasma total cholesterol, HDL-cholesterol and LDL-cholesterol were detected.

**Table 2:** Relative leukocyte telomere lengths in AscAA patients and healthy controls. Risks for aneurysms are estimated based on controls.

	Relative LTL	AscAA cases	Control cases	Adjusted OR (95% CI)#	P-value	Aneurysm size >5 cm
Mean LTL (SD)		0.87 (0.30)	0.61 (0.20)		<0.001*	
*By quartiles:*						
First quartile	0.18–0.57	12 (14%)	32 (37%)	–	–	5
Second quartile	0.58–0.70	18 (21%)	28 (33%)	3.6 (1.1–11.5)	<0.05	11
Third quartile	0.71–0.88	21 (24%)	19 (22%)	5.6 (1.7–18.9)	<0.01	13
Fourth quartile	0.89–1.63	35 (41%)	7 (8%)	23.3 (5.6–97.3)	<0.001	22
*By median:*						
Short	<0.70	30 (35%)	60 (70%)	–	–	16
Long	0.70<	56 (65%)	26 (30%)	4.9 (2.1–11.3)	<0.001	35

*LTL,* leukocyte telomere length, *AscAA,* ascending aortic aneurysm, *SD,* standard deviation, *OR,* odds of ratio, *CI,* confidence interval,

# adjusted *by age, gender, HDL-cholesterol, triglycerides, hypertension and CHD.*

* *independent samples t-test.*

Logistic regression analysis was carried out in the studies of association between relative LTL and the risk of AscAAs (Table 2). When relative LTL results were divided into two groups based on the median of LTLs (T/S ratio 0.70), individuals with longer telomeres had a significantly higher risk for aneurysms (age, gender, HDL-cholesterol, triglycerides, hypertension and CHD adjusted OR 4.9, 95% CI 2.1–11.3, p<0.001) compared with individuals with shorter telomeres. Furthermore, the results of the relative LTL were divided into 4 groups according to the telomere lengths in the whole data and a progressively higher risk for AscAAs was observed in the patient group with the longer leukocyte telomeres. The first quartile (shortest telomeres) was used as the reference group and the adjusted ORs for second, third and fourth quartiles were 3.6 (95% CI 1.1–11.5, p<0.05), 5.6 (95% CI 1.7–18.9, p<0.01) and 23.3 (95% CI 5.6–97.3, p<0.001) (age, gender, HDL-cholesterol, triglycerides, hypertension and CHD adjusted), respectively. The same differences were seen when relative LTL results were divided into tertiles or quintiles.

No correlation was found between aneurysm size and relative LTL (p = 0.184). However, patients with an AscAA diameter larger than 5 cm at the aortic root belonged to the longer telomere group (Table 2).

### Quantitative RT-PCR Analyses of Telomerase

The study samples used for RT-qPCR analysis were from individuals whose mean ages were 60 years in the AscAA group and 64 in the control group. The expression of hTERT was elevated in the samples of the AscAA patients (ΔΔCt 0.071) as compared with the controls (ΔΔCt 0.013) (p<0.05).

### Immunostaining of Aortic Tissues with Telomerase Antibody

**Figure 1:**
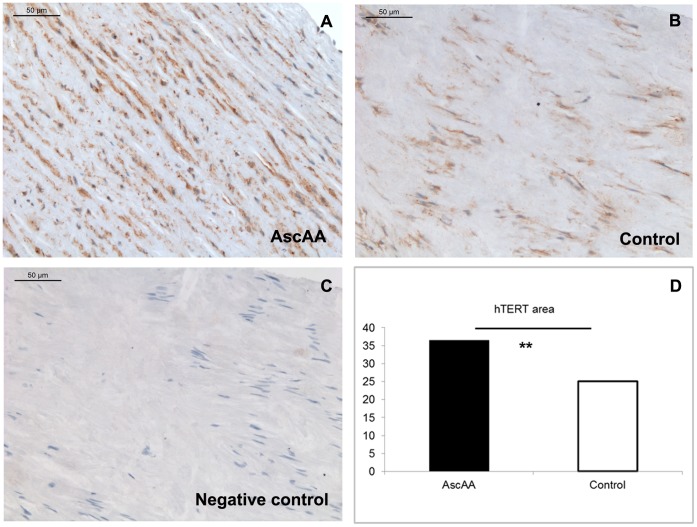
Immunostaining of hTERT in A) AscAA, B) control aorta and C) negative control slides (original magnification×20). Quantified immunostained areas of hTERT protein in AscAA and control aorta slides are presented in a bar chart (D). Statistically significant differences between the groups are marked as **(p<0.01). *AscAA*, ascending aortic aneurysm, *hTERT*, telomerase.

In the aortic tissue samples, the mean age of the AscAA patients was 57 years, for the controls it was 69 years (p<0.05). Figure 1 displays the immunostaining of the AscAA and control tissue sections. Quantitative analysis of the immunostained areas in the aortic tissue slides from AscAAs patients showed elevated levels of hTERT protein when compared with the control aortic tissue slides (relative area of 36.5 for AscAA vs. 25.0 for controls, p<0.01).

## Discussion

In our study, patients with AscAAs had significantly longer telomeres than their healthy age matched controls i.e. long telomeres were associated with a high risk of AscAAs. Furthermore, there was a trend towards longer telomeres in the DNA samples of AscAA patients with an aneurysm larger than 5 cm at the aortic root. Relative LTL values were associated with age and in our study, age and relative LTL displayed a strong inverse correlation.

An earlier study of Atturu et al. reported shorter telomere lengths in white blood cells of AAA patients [10]. Similarly, Wilson et al. have described shorter telomeres in blood leukocytes and aortic tissues and Cafueri et al. in endothelial cells, SMCs, keratinocytes and blood lymphocytes of AAA patients compared with controls [12], [16]. Shorter telomeres have been measured also in the patients with aortic dissections [17]. Today, there are two studies showing shortened telomeres in AscAAs [11], [18]. Blunder et al. measured shortened telomeres in cultured SMCs of thoracic aortic aneurysm vessel wall [18]. Prior to our analysis, examinations of blood LTLs in AscAA have been carried out in the preliminary study by Balistreri et al., where the telomere restriction fragment method was used to analyse 10 AscAA samples [11]. The results of our blood LTL study are opposite to the results of Balistreri et al., but this may be due to the different methods and sample sizes being used. Patients with AscAA can be divided into clinical populations according to age at the time of surgery and those with a positive family history of aneurysms [19]. Accordingly, we cannot be sure that our AscAA patients represent the same clinical type of thoracic aortic aneurysms as the patients examined in the study of Balistreri et al. where 3 aneurysmal phenotypes were characterized among 161 patients but no exact information about the phenotypes of those 10 studied samples was provided [11]. Some forms of aneurysms are associated with the systemic aged phenotype, whereas our results indicate that in the case of AscAAs, the aged phenotype is not inevitably associated with aneurysms. It seems to be clear that there is an optimal telomere length to maintain cell stability and apoptosis; too short or too long telomere length may contribute to aneurysm formation once the critical equilibrium is disturbed. Furthermore, longer telomeres do not mean a longer lifespan, in fact it is still a mystery how these differences in telomere length can affect an individual’s lifespan. Telomere shortening will occur in every individual, although shortening and elongation can vary over time. The individuals who are born with longer telomeres experience the largest telomere erosion during their lifetime [20]. However, there are studies which have detected no correlation between telomere length and mortality in general [21], [22]. LTLs can also vary in different blood cell types and this might account for the finding of longer LTL results of AscAAs compared with previous reports. Cells with longer telomeres may delay the development of cell senescence causing chromosomal instability and genetic abnormalities [23], [24], and further, it seems to place these individuals at a higher risk for suffering AscAAs.

We also studied the expression of hTERT at both in mRNA and protein level from the tissue samples of AscAA and control aorta. hTERT mRNA expression was elevated in aortic wall samples of AscAAs when compared with the controls. Furthermore, the immunostaining of the aortic tissue samples with hTERT antibody revealed more hTERT protein in the tissue sections of AscAA patients than in controls. In the immunostaining study the age of AscAA patients and controls differed but the results were similar to those obtained in the mRNA studies which were well matched in terms of age. This problem with different ages is very commonly encountered in aortic aneurysm studies, as it is challenging to obtain tissue samples from young healthy aorta [18]. Our control tissue samples were taken during coronary bypass operations and these tissues may have exhibited atherosclerosis. There are several reports of an association between shorter blood leukocyte telomere lengths and atherosclerotic cardiovascular disease [25], [26]. However, Gizard et al. claimed that the expression of telomerase is activated during atherosclerosis [27] and therefore, our finding of elevated telomerase in AscAA tissues should not be attributable to the used control specimens. Previously, low hTERT expression has been found in the samples of abdominal aortic aneurysms (AAA) [28]. Since we found opposite results in our study, this suggests that the phenotype of AscAA wall differs from the phenotype of AAA wall. Ascending aorta and abdominal aorta are formed from different origins during embryogenesis [29] and there are differences in the structure of the media layer between these two sites [30]. Abdominal aorta is also much more vulnerable to atherosclerotic changes compared with ascending aorta [31].The main function of hTERT is to add back telomeric repeats and high hTERT expression may act as a protective mechanism against aneurysm formation, and consequently, this leads to longer telomeres.

In our previous study [15], we have shown that OPN, MMP-2 and MMP-9 protein and mRNA levels were significantly higher in the plasma and tissue samples from AscAA patients compared with controls. MMP-9 and MMP-2 are proteolytic enzymes that are overexpressed in the enlargement of the aorta. It has been shown that downregulation of MMP-9 in glioma cells reduces the levels of proteins which play a direct role in regulating telomere length [32]. Furthermore, in their study Findeisen et al. found that TERT could induce MMP-2 transcription [33]. The same study noted that TERT deficiency attenuated AAA formation, and furthermore, decreased MMP-2 activity. This is in line with our studies of elevated MMPs and hTERT in the tissues of AscAA. In addition, longer LTLs were found in the blood samples of AscAA patients. These results indicate that changes in the levels of MMP-9, MMP-2 and hTERT might be mediated through the same genetic mechanism.

Our study was based on the blood LTL and not on the actual aortic tissue telomere length. According to Wilson et al., there is a strong correlation between abdominal aortic tissue and blood leukocyte telomere content [12]. Cafueri et al. have also shown the same systemic nature of telomere lengths in different tissues of AAA patients [16]. Moreover, the studies of Wilson et al., Cafueri et al. and Friedrich et al. justify the use of blood leukocyte telomere length as a predictor of the state of other tissues since there seems to be a genetic mechanism controlling telomere lengths throughout the body [12], [16], [34]. Studies with blood leukocytes are also more practical to implement than studies with aortic wall tissues. However, it remains an open question whether longer telomeres are a cause or a consequence of AscAAs.

This study demonstrates the role of telomere length and levels of hTERT in AscAAs. We found longer relative blood LTLs in the AscAA samples compared with their age and sex–matched controls. In addition, we observed elevated expression of hTERT protein in the samples of AscAAs, and furthermore, elevated mRNA expression in the tissue samples of AscAAs. Further studies will be needed to determine whether relative blood LTL can be used in the estimation of the risk for AscAAs in sporadic and familial cases, for monitoring the progression of AscAA enlargement or even for aiding physicians in their therapeutic decisions, including decisions of the timing about aortic surgery.
